# PB.15: Pain in mammography: where and why does it arise?

**DOI:** 10.1186/bcr3515

**Published:** 2013-11-08

**Authors:** D O'Leary, Z Al Maskari

**Affiliations:** 1 University College Dublin, Ireland

## Background

The purpose of the study was to determine the intensity of pain experienced by women undergoing mammography examination through investigation of biological, psychological and technical factors that influence any pain felt during the examination.

## Methods

Sixty-four women presenting for diagnostic and screening mammography were examined. Pain experience data were collected at three discrete time-points during mammography using both a visual analogue and Likert scales.

## Results

Pain due to compression was rated by 96.6% of women. The pain/discomfort averaged between mild (42%) to moderate (49%) in the craniocaudal (CC) projection and between mild (22%), moderate (61%) to very severe pain (13.5%) in the mediolateral oblique (MLO) projection. Correlation identified significant associated factors for pain: women's age (*P *= 0.001), menstruation (*P *= 0.042), menopausal status (*P *= 0.002) and marital status (*P *= 0.000). A unique aspect was the investigation of the sources of pain in each projection. Pain experienced during breast compression mainly arises from compression pressing on the middle of the breast and chest wall in the CC and from the compression pressing on the sternum, middle and underside of the breast and the axilla in the MLO.

## Conclusion

This study supports other published work that women experience pain during breast compression within mammography and that the radiographer plays a major role in the women's experience of pain. The study contributes to the pain debate by showing where the pain arises in the breast specifically during compression and the degrees of pain felt.

